# Factors Influencing Specialty and Training Center Choices Among Saudi Medical Residents

**DOI:** 10.7759/cureus.48844

**Published:** 2023-11-15

**Authors:** Mohammed A Sindi, Mahmoud H Almadani, Marah A Sindi, Ali H Alturaif, Khaled W Altahini, Naif Aljohani, Sary J Zaher, Ahmad H Alhibshi, Nidal H Bokhary, Faisal Alfaidi, Khalid Alfaidi, Maisa Al-Sebaei, Maha R Alshehri

**Affiliations:** 1 General Dentistry, King Abdulaziz University Hospital, Jeddah, SAU; 2 Department of Neurology, King Abdulaziz University Hospital, Jeddah, SAU; 3 Department of Family Medicine, Ministry of Health, Dammam, SAU; 4 Department of Physical Medicine and Rehabilitation, Prince Sultan Military Medical City, Riyadh, SAU; 5 Department of Psychiatry, Erada Mental Health Complex, Tabuk, SAU; 6 Department of Family Medicine, Ministry of Health, Makkah, SAU; 7 Department of Family Medicine, Ministry of Health, Jeddah, SAU; 8 Department of Internal Medicine, King Abdulaziz University Hospital, Jeddah, SAU; 9 Department of Emergency Medicine, King Abdulaziz University Hospital, Jeddah, SAU; 10 Department of Radiology, King Abdulaziz Medical City Jeddah, Jeddah, SAU; 11 Department of Urology, King Abdullah Medical Complex, Jeddah, SAU; 12 Oral and Maxillofacial Surgery, King Abdulaziz University Hospital, Jeddah, SAU; 13 Pediatric Dentistry, King Abdulaziz University Hospital, Jeddah, SAU

**Keywords:** factors, training, medical residents, training center choice, speciality choice, cross-sectional study, medical education

## Abstract

Aim: This cross-sectional study aims to identify and quantify the factors influencing Saudi medical residents in selecting their desired specialty and primary training center, while examining the associations between these factors.

Methods: The study received approval from an institutional ethical committee at King Abdulaziz University. An electronic questionnaire was designed and validated via content, face validity, and the Content Validity Index. The sample size was calculated based on a 95% CI and a 5% margin of error. The study targeted all current residents in the current Saudi Specialty Certificate Programs. Descriptive statistics summarized demographic characteristics, training-related information, and factors influencing the selection of a specialty and training center. Fisher's exact test and Chi-square tests were employed for data analysis.

Results: A total of 387 respondents completed the survey, with a 32.3% response rate. The majority of respondents were male (n = 232, 59.9%), and the majority were also married (n = 67.2%), with internal medicine (n = 92, 23.8%) and family medicine (n = 74, 19.1%) being the most prevalent specialties. Notably, 89.4% (n = 346) reported matching into their first-choice specialty, and 67.2% (n = 260) into their first-choice training center. Furthermore, 90.2% (n = 349) had prior training (elective/internship) in their chosen specialty, and 63% (n = 244) had previous training (elective/internship) at their primary training center. Prior exposure to both specialty and center significantly resulted in them being the resident’s top choice (p < 0.01). Multiple factors influencing the choice of either the specialty or the center were found to have statistically significant associations with the gender, specialty, residency level, sector of the training center, and timing of the specialty decision (p < 0.05).

Conclusion: This study reveals the substantial influence of early experiences on Saudi medical residents' specialty and training center choices. It also uncovers gender disparities and variations in the influence of specialty-related factors. Future research with larger and more diverse samples is recommended to gain a deeper understanding of the multifactorial decision-making processes, enabling the development of strategies to better meet the evolving needs and preferences of healthcare professionals in Saudi Arabia.

## Introduction

The journey to becoming a specialized medical professional is characterized by a series of critical decisions, none more profound than the selection of one's medical specialty and the choice of a training center for residency. This pivotal crossroad in the career of medical residents is influenced by a myriad of factors, each of which plays a significant role in shaping their professional trajectory [1,2]. Understanding the motivations and decision-making processes behind these choices is not only crucial for medical educators and program directors but also offers valuable insights into the evolving landscape of healthcare.

The choice of a medical specialty, among the most impactful decisions a medical graduate makes, encompasses a complex interplay of personal passion, professional aspirations, and practical considerations. The question of why certain individuals chooses specific fields of medicine over others and at what point in their educational journey this decision crystallizes has been the subject of extensive research and debate [3-10]. Among the most prominent influential factors observed in the literature are financial stability or career opportunities [7,9,11], work-life balance [4,7,8], and creativity or challenges associated with specialty [4,5,11]. However, the majority of prior research in this field has predominantly concentrated on medical students, often encompassing those in their early years of medical education who may not have had the opportunity to experience clinical specialties [3-15].

In parallel, the selection of a training center for residency is another significant milestone. Residents must consider a variety of factors, including the quality of training, geographic location, the reputation of the institution, and financial considerations, when making this decision [16,17]. While the stipend/salary paid for medical residents in Saudi Arabia is usually dependent on their primary training center acting as a sponsor, there’s a notable scarcity of research exploring the impact of personal reasons on the choice of training center, despite its potential decisive importance.

Inarguably, the selection of a medical specialty and training center is a complex decision influenced by a multitude of factors. However, existing research, often reliant on data from medical students yet to experience clinical specialties, raises concerns about the reliability of its findings. Furthermore, the dearth of literature pertaining to the choice of primary training centers highlights the need for further exploration. This study aims to bridge these knowledge gaps by retrospectively identifying and quantifying the factors shaping the choices of medical residents in their specialty and primary training center while also examining the interplay between these factors, including gender, specialty, prior training experiences, and sector (such as governmental, private, or military), to provide a more comprehensive understanding of the intricate decision-making processes guiding the careers of medical professionals.

## Materials and methods

Study protocol and sample criteria

The protocol of this questionnaire-based, cross-sectional study received approval from the Research Ethics Committee at the Faculty of Dentistry (REC-KAUFD), King Abdulaziz University, Saudi Arabia (approval no.: 094-04-23, dated: August, 2023). Subsequently, the questionnaire was made accessible through diverse online platforms, and data collection was halted on October 1, 2023, coinciding with the commencement of the new academic year for Saudi Specialty Certificate medical residencies in Saudi Arabia. Saudi Specialty Certificate medical residencies are 26 in total and range from 3 to 7 years, with one or more certifying exams that earn the resident the rank of “specialist” in the Saudi Commission for Health Specialties.

The sample size calculation for this study was carried out with a 95% confidence interval and a 5% margin of error, targeting a population of 10,150 medical residents. The determination of this population size was based on the historical data of applicants who have undertaken the Saudi Specialty Certificate over the last four years, which reflects the average duration of Saudi Specialty Certificate residency programs. The data was sourced from publicly available information provided by the Saudi Commission for Health Specialties [18]. Utilizing this information, we used an online sample size calculator (RaoSoft) to compute the estimated required sample size, which amounted to 371 medical residents. This sample size was considered suitable to provide statistically reliable results.

The inclusion criteria for this study encompassed Saudi Specialty Certificate medical residents, with the exclusion of those who had completed their training. Specifically, residents specializing in fields related to medicine and surgery were included in the research. Conversely, residents in dentistry, laboratories, medical technology, pharmacists, pharmacy technicians, as well as technicians and health assistants, were excluded from the study based on the specified criteria.

Questionnaire development and validation

An electronic questionnaire was designed for data collection, consisting of a digitally obtained consent form followed by four sections. For the context of this research, we focused primarily on two sections: demographics and training-related information, with all questions and responses available in Appendix. The consent form included three vital declarations: the participant's confirmation of their current status as a medical resident in an accredited Saudi Specialty Certificate training program, their understanding that no identifying information would be collected to ensure response anonymity, and their awareness of the right to withdraw from the study without incurring any penalties or consequences.

The questionnaire underwent a meticulous validation process to ensure its reliability and relevance. Initially, it was reviewed by a panel of three seasoned professionals, each with over a decade of experience in key roles across various Saudi Arabian academic institutions. Their feedback proved highly satisfactory, necessitating only minor linguistic adjustments. For comprehensive content validation, the refined questionnaire was then assessed by two distinct subject-matter experts. Each questionnaire item was evaluated for relevance to the intended construct and overall clarity. Content validation was quantified using the scale-level content validity index based on proportion relevance (S-CVI/Ave) and the scale-level content validity index based on the universal agreement method (S-CVI/UA) [19,20]. The S-CVI/Ave and S-CVI/UA for relevance and clarity were 0.96 and 0.92, and 0.96 and 0.92, respectively, indicating that the scale of our questionnaire achieved satisfactory levels of content validity.

Data management and statistical analysis

Data cleaning, coding, and all statistical analyses were conducted using IBM Corp. Released 2022. IBM SPSS Statistics for Windows, Version 29.0. Armonk, NY: IBM Corp.

Descriptive statistics were used to summarize both demographic variables and questions pertaining to training. Frequencies and percentages were used to present the distribution of responses for most variables, while age was summarized using means and standard deviations. To assess whether the choice of specialty or primary training center was associated with any prior training, a Fisher’s exact test was used. To explore potential significant associations with factors influencing choices of specialty and primary training center, two independent variables were considered: factors influencing the choice of medical specialty and factors influencing the choice of primary training center. To assess these associations, a Chi-square analysis was conducted in the following manner: Z scores were initially computed for all independent variables interacting with the demographic variables. Subsequently, Chi-square analysis was performed for all Z scores using the specific degree of freedom determined for each dataset. To account for any potential Type I errors, Bonferroni-corrected P-values were calculated for each dataset, with a pre-correction alpha level of 0.05 serving as the threshold for establishing statistical significance. This analytical approach was followed to examine the relationships and directionality of all statistically significant interactions between the chosen independent variables and demographic characteristics.

## Results

The questionnaire was distributed to approximately 1,200 medical residents, and a total of 387 residents consented to participate and successfully completed the questionnaire, resulting in a response rate of 32.3%. This response rate exceeded the minimum estimated sample size of 371 residents, validating the adequacy of the dataset. Among the participants, the majority were male (n = 232, 59.9%), with a mean age of 27.85±2.51 years. A significant portion of respondents reported being married (n = 260, 67.2%). Regarding their medical specialties, most residents were enrolled in internal medicine (n = 92, 23.8%) and family medicine (n = 74, 19.1%) residency programs. Additional demographic details can be found in Table 1.

**Table 1:** Descriptive statistics of the sample characteristics

Variable		Count	Percentage
Gender	Male	232	59.9%
	Female	155	40.1%
Marital status	Single	260	67.2%
	Married	120	31%
	Divorced/widowed	7	1.8%
Current specialty program	Internal medicine	92	23.8%
	Family medicine	74	19.1%
	Neurology	30	7.8%
	Emergency medicine	26	6.7%
	Rehabilitation medicine	25	6.5%
	Pediatrics	23	5.9%
	Diagnostic radiology	20	5.2%
	Psychiatry	18	4.7%
	General surgery	18	4.7%
	Adult critical care medicine	11	2.8%
	Neurosurgery	11	2.8%
	Preventive medicine	10	2.6%
	OB/GYN	6	1.6%
	Orthopedic surgery	5	1.3%
	Dermatology	4	1%
	Ophthalmology	3	0.8%
	Anesthesia	3	0.8%
	Plastic surgery	2	0.5%
	Pediatric neurology	2	0.5%
	ENT	1	0.3%
	Urology	1	0.3%
	Forensic medicine	1	0.3%
	Pediatric surgery	1	0.3%
Current residency level	R1	117	30.2%
	R2	132	34.1%
	R3	79	20.4%
	R4	45	11.6%
	R5	14	3.6%

The majority of participants indicated they matched into their first-choice specialty (n = 346, 89.4%) and primary training center (n = 260, 67.2%). Additionally, a significant number of residents reported having prior training in their chosen specialty (n = 349, 90.2%) and the primary training center where they were currently enrolled (n = 244, 63%). When asked about the factors influencing their choice of specialty, a substantial proportion of participants cited their preference for the clinical or surgical aspects of the field (n = 158, 40.8%) or previous training experiences (n = 158, 40.8%). Similarly, factors influencing the choice of their training center were primarily related to the center's strong reputation for training programs (n = 92, 23.8%) and prior training experiences at the center (n = 66, 17.1%). Additional descriptive statistics regarding training-related questions can be found in Table 2.

**Table 2:** Descriptive statistics of the training-related questions KFSHRC: King Faisal Specialists Hospital & Research Center; MOH: Ministry of Health; MNGHA: Ministry of National Guard Health Affairs

Variable		Count	Percentage
The sector of my primary training center is…	MOH, Medical Cities, and KFSHRC	195	50.4%
	Ministry of Education (University Hospital)	89	23.0%
	Military (Armed Forces Hospital)	59	15.2%
	MNGHA	31	8.0%
	Private	13	3.4%
I did some training/elective/internships in my current specialty before matching	Yes	349	90.2%
	No	38	9.8%
My current specialty was my first choice during matching	Yes	346	89.4%
	No	41	10.6%
I did any training/elective/internships in my current center before matching	Yes	244	63.0%
	No	143	37.0%
My current center was my first choice during matching	Yes	260	67.2%
	No	127	32.8%
I decided on pursuing my current specialty…	Before medical school	14	3.6%
	During medical school	148	38.2%
	During internship	161	41.6%
	After internship (during attachment/service)	64	16.5%
The most-influential factor in my choice of specialty was...	Because I like the clinical/surgical practice of my specialty	158	40.8%
	Because of previous training	96	24.8%
	Future financial stability (anticipated salary or ease of acquiring a future job)	59	15.2%
	Ease of training	26	6.7%
	Ease of acceptance	14	3.6%
	Influenced by family or friends	14	3.6%
	Because I was matched into it (not my first choice)	12	3.1%
	Due to personal conditions (i.e., diseases in the family)	3	0.8%
	Other	5	1.3%
The most-influential factor in my choice of my center was...	Because the center is reputed to have a strong training	92	23.8%
	I had previous practice at this center	66	17.1%
	Because I was matched into it (not my first choice)	61	15.8%
	The center is close to where I live	43	11.1%
	Because of previous personal experience at the center	28	7.2%
	My family/friends are training/practicing at this center	23	5.9%
	The center provides sponsorship contracts	22	5.7%
	Because I took my medical school education at this center	17	4.4%
	Because the center has a large number of seats	17	4.4%
	Because the center promises future jobs/training (fellowships) to its graduate	12	3.1%
	Because the center is reputed to have an easy training	5	1.3%
	Other	1	0.3%

Fisher's exact test was utilized to explore the influence of prior training in a specific specialty or center on medical residents' choices for their specialty and primary training center during their residency. The analysis established statistically significant associations between prior training and both specialty and training center selection (p < 0.01). Further examination using Chi-square tests revealed notable findings related to the choice of specialty and training center.

Regarding specialty choices, it was found that more female residents were matched into specialties other than their first choice (χ² = 3.108, p < 0.002), and a similar observation was seen among pediatric surgery residents, who were less likely to select their specialty as their first choice (χ² = 5.597, p < 0.0001). Family medicine residents expressed a distinct preference for their specialty due to the perceived ease of training compared to other specialties (χ² = 5.694, p < 0.0001). Additionally, residents in year five training level displayed a stronger inclination towards their specialty because of their preference for its clinical and surgical aspects (χ² = 3.481, p < 0.0001). In contrast, residents from MOH (Ministry of Health), medical cities, and KFSHRC (King Faisal Specialists Hospital & Research Center) appeared less inclined to choose their specialty based on clinical or surgical aspects (χ² = -3.436, p < 0.001). Residents who made their specialty choice while still medical students favored the clinical and surgical aspects of their chosen field (χ² = 4.166, p < 0.0001), while those who decided during their internship or service were significantly less likely to be matched into their preferred specialty (χ² = 4.748, p < 0.0001).

Regarding the choice of training center, residents from MOH, medical cities, and KFSHRC were significantly more likely to select their center due to its proximity to their place of residence (χ² = 4.96, p < 0.001) or because of its higher ease of acceptance (χ² = 3.688, p < 0.001). Conversely, fewer residents from university hospitals opted for their center based on its proximity to their residence (χ² = -3.417, p < 0.001), but a greater number chose it because they had received their medical education there (χ² = 5.358, p < 0.0001).

## Discussion

In this cross-sectional study, our primary aim was to investigate the factors guiding the choices of current Saudi medical residents regarding both their selected medical specialties and their preferred training centers, all while considering potential associations with demographic variables. Additionally, we explored whether prior exposure to specific specialties or training centers influenced a resident's ultimate decision.

Our analysis revealed a noteworthy observation among Saudi medical residents, with a substantial proportion selecting both their specialty (90.2%) and training center (63%) based on prior training experience, whether during or after their medical school. We identified a significant correlation between this prior exposure and the likelihood of the chosen specialty or center becoming the resident's first preference.

In Saudi Arabia, while variations exist among schools, medical students commonly undertake a mandatory one-month elective training during their medical education. Furthermore, many institutions offer interns an average of six months during which they have the freedom to choose their preferred specialty and training center. Given these circumstances, we initially hypothesized that a considerable number of residents would have prior training in their first-choice specialty and center. Surprisingly, the figures exceeded our expectations. Particularly noteworthy is the fact that 41.8% of residents made their choices during medical school or even earlier, suggesting a prevailing trend among Saudi students to adhere to their specialty selections made during this period. This underscores the need for curricula that expose medical students to a diverse array of specialties before graduation. Supporting this notion is a study by Alkhaneen et al., where over half of their sample chose emergency medicine due to prior experience gained through hospital rotations [21]. This contrasts with findings from Singh (2019), who observed a tendency among UK junior doctors to change their specialty preferences [22]. This difference could be attributed to the UK's requirement for doctors to spend up to two years as junior doctors before becoming eligible to apply for specialization, while Saudi residents can apply for specialization very early during their internship, provided they meet the necessary requirements.

Interestingly, we found a significant disparity in residency matching outcomes for females, with a significantly larger proportion failing to secure their first-choice specialty. While previous studies have delved into the impact of gender on medical specialty selection, these studies primarily focused on the preferences of medical students rather than evaluating the consequences of residency matching, where residents may not obtain their preferred specialty [2,3,15,23]. This mismatch may be attributed to a divergence in the aspirations of females and males. Our limited, cross-sectional sample reveals that more females are currently residents in highly competitive fields like obstetrics and gynecology (OB/GYN) and pediatrics, while a greater number of males in our limited sample gravitated towards less competitive specialties such as preventive medicine, adult critical care medicine, and anesthesia. Our analysis also noted a decreased likelihood of pediatric surgery residents securing their first-choice specialty, but this observation may be unreliable due to the limited number of respondents from this specialty in our questionnaire.

Family medicine residents exhibited a notably higher preference for their specialty compared to residents in other fields, driven by the perceived ease of training when compared to other specialized disciplines. Notably, Saudi Arabia's family medicine residency program has the largest number of available seats and training centers, a feature exclusive to this specialty. An additional factor contributing to the appeal of family medicine is the recent reduction of the program's duration from four years to three, with most of the initial year primarily dedicated to rotations in other specialties. It's important to acknowledge that it was not possible in our study to delve into the specific practice breakdown of family medicine residents compared to those in other specialties. However, the shorter training period and the diverse exposure to various medical areas may have played a pivotal role in the perceived ease of this specialty's training. Residents specializing in Saudi Specialty Certificate programs requiring five years or longer, such as general surgery, neurosurgery, plastic surgery, orthopedic surgery, ear, nose, and throat (ENT), cardiac surgery, urology, obstetrics and gynecology (OBGYN), and anesthesia, were more likely to express a preference for their specialty based on genuine passion for the practice. These specialties, historically associated with reduced work-life balance [24-27], tend to attract residents who prioritize the inherent rewards of the field.

In Saudi Arabia, a resident's salary is typically covered by the sponsor of their primary training center. In recent years, several sponsors have decreased the number of contracts available to residents. However, a few institutions, including the Ministry of Health (MOH) hospitals, medical cities, and the King Faisal Specialists Hospital & Research Center (KFSHRC), have continued to offer contracts to all their residents. Interestingly, our study revealed that residents of these hospitals were the least likely to choose their specialty solely based on the appeal of its practice. This suggests that the availability of paid training contracts in these centers may have influenced their choice. Simultaneously, due to the abundance of MOH-affiliated facilities, residents often selected these centers due to their proximity to their place of residence. In contrast, residents of university hospitals were less likely to prioritize proximity when choosing their training center. This is likely attributed to the same residents selecting university hospitals more frequently because they had received their medical education there, underscoring the influence of educational and institutional ties on their decisions.

Nonetheless, it's important to acknowledge some potential shortcomings in our study. First, our findings are based on self-reported recalled data from a limited sample of Saudi medical residents, and individual perceptions and experiences can vary widely. Second, the limited number of respondents from certain specialty programs might have affected the robustness of our conclusions in those areas. Additionally, the cross-sectional nature of our study captures a single point in time, which might not fully represent the dynamic nature of residents' career choices. Despite these limitations, our research provides valuable insights into the factors influencing the choices of medical residents in Saudi Arabia and underscores the need for further investigation in this field.

## Conclusions

Our study underscores the significant impact of early experiences on the choices of Saudi medical residents in terms of specialty and training center selection while also revealing notable associations with factors such as gender, timing of career decisions, and the specific sector of training centers. To advance our understanding of these complex dynamics, we recommend future research with larger sample sizes and greater diversity in terms of specialty choices, fostering increased variability in the analysis. Such expanded investigations will provide a more comprehensive insight into the multifactorial decision-making processes of medical residents and contribute to the development of strategies aimed at optimizing their training and career choices to better serve the evolving needs and preferences of healthcare professionals in the country.

## Appendices

**Table 3:** Sections 1, 2 of the validated questionnaire KFSHRC: King Faisal Specialists Hospital & Research Center; MOH: Ministry of Health; MNGHA: Ministry of National Guard Health Affairs

Question	Responses
Gender	Male
	Female
Age (in years)	(open ended)
Current specialty program	Adult critical care medicine
	Anesthesia
	Dermatology
	Diagnostic radiology
	Emergency medicine
	ENT
	Family medicine
	Forensic medicine
	General surgery
	Internal medicine
	Neurology
	Neurosurgery
	OBGYN
	Ophthalmology
	Orthopedic surgery
	Pediatric neurology
	Pediatric surgery
	Pediatrics
	Plastic surgery
	Preventive medicine
	Psychiatry
	Rehabilitation medicine
	Urology
Current residency level	R1
	R2
	R3
	R4
	R5
Where is your primary training center located	Central region
	Eastern region
	Western region
What is the sector of your primary training center	Military (Armed Forces)
	Ministry of Education (University Hospital)
	MNGHA
	MOH, Medical Cities, and KFSHRC
	Private
Marital status	Divorced/widowed
	Married
	Single
Was your current specialty your first choice?	Yes
	No
Was your current primary training center your first choice?	Yes
	No
At which point did you decide on pursuing your current specialty?	After internship (during attachment/service)
	Before med school
	During internship
	During med school
Did you do any training/elective/internships in your current specialty before matching? (during or after med school)	Yes
	No
Did you do any training/elective/internships in your current primary training center before matching? (during or after med school)	Yes
	No
What influenced your choice in specialty the most?	Because I like the clinical/surgical practice of my specialty
	Because of previous training (electives/internships/service)
	Future financial stability (anticipated salary or ease of acquiring a future job)
	Ease of training (during residency/fellowship)
	Ease of acceptance (during matching)
	Influenced by family or friends
	Because I was matched into it (not my first choice)
	Due to personal conditions (i.e., diseases in the family)
What influenced your choice in your center the most	Because the center is reputable to have a strong training
	I had previous practice (electives/internships) at this center
	Because I was matched into it (not my first choice)
	The center is close to where I live
	Because of previous personal experience at the center
	My family/friends are training/practicing at this center
	The center provides sponsorship contracts
	Because I took my medical school education at this center
	Because the center has a large number of seats (easy acceptance)
	Because the center promises future jobs/training (fellowships) to its graduate
	Because the center is reputable to have an easy training

## References

[1] Al-Ansari SS, Khafagy MA (2006). Factors affecting the choice of health specialty by medical graduates. J Family Community Med.

[2] Heikkilä T, Hyppölä H, Kumpusalo E (2011). Choosing a medical specialty--study of Finnish doctors graduating in 1977-2006. Med Teach.

[3] Abdulghani HM, Al-Shaikh G, Alhujayri AK (2013). What determines the selection of undergraduate medical students to the specialty of their future careers?. Med Teach.

[4] Al-Zubi M, Ali MM, Alzoubi S (2021). Preference of and factors that influence future specialty among medical students in Jordan: A cross-sectional study. Ann Med Surg (Lond).

[5] Al-Fouzan R, Al-Ajlan S, Marwan Y, Al-Saleh M (2012). Factors affecting future specialty choice among medical students in Kuwait. Med Educ Online.

[6] Chang P-Y, Hung C-Y, Wang K-l (2006). Factors influencing medical students' choice of specialty. Jr For Med Assoc.

[7] Correia Lima de Souza L, Mendonça VR, Garcia GB, Brandão EC, Barral-Netto M (2015). Medical specialty choice and related factors of Brazilian medical students and recent doctors. PLoS One.

[8] Clarke N, Crowe S, Humphries N, Conroy R, O'Hare S, Kavanagh P, Brugha R (2017). Factors influencing trainee doctor emigration in a high income country: a mixed methods study. Hum Resour Health.

[9] Dikici MF, Yaris F, Topsever P, Tuncay Muge F, Gurel FS, Cubukcu M, Gorpelioglu S (2008). Factors affecting choice of specialty among first-year medical students of four universities in different regions of Turkey. Croat Med J.

[10] Ie K, Murata A, Tahara M, Komiyama M, Ichikawa S, Takemura YC, Onishi H (2020). Relationship between medical students' career priority and specialty choice: A nationwide multicenter survey. J Gen Fam Med.

[11] Asiri WM, Shati AA, Alrowaibah NA, Althumairi RK, Alqahtani GM, Mahmood SE (2023). The influencing factors of choosing future medical specialties among students in Saudi Arabia: A nationwide multicenter survey. Medicine (Baltimore).

[12] Khamees A, Awadi S, Al Sharie S, Faiyoumi BA, Alzu'bi E, Hailat L, Al-Keder B (2022). Factors affecting medical student's decision in choosing a future career specialty: A cross-sectional study. Ann Med Surg (Lond).

[13] McDonald C, Henderson A, Barlow P, Keith J (2021). Assessing factors for choosing a primary care specialty in medical students; A longitudinal study. Med Educ Online.

[14] Mohamed EY (2022). Specialty preferences and factors affecting the choices of postgraduate specialty among undergraduate medical students. Pak J Med Sci.

[15] Ng-Sueng LF, Vargas-Matos I, Mayta-Tristán P (2016). Gender associated with the intention to choose a medical specialty in medical students: a cross-sectional study in 11 countries in Latin America. PLoS One.

[16] Aagaard EM, Julian K, Dedier J, Soloman I, Tillisch J, Pérez-Stable EJ (2005). Factors affecting medical students' selection of an internal medicine residency program. J Natl Med Assoc.

[17] Mayer KL, Perez RV, Ho HS (2001). Factors affecting choice of surgical residency training program. J Surg Res.

[18] SCFHS SCFHS (2023). SCFHS: Residency programs public data: Saudi Commission for Health Specialties. https://public.scfhs.org.sa/SCFHS_Residency_Programs.html.

[19] Polit DF, Beck CT (2006). The content validity index: are you sure you know what's being reported? Critique and recommendations. Res Nurs Health.

[20] Yusoff MSB (2019). ABC of content validation and content validity index calculation. Edu Med Jr.

[21] Alkhaneen H, Alhusain F, Alshahri K, Al Jerian N (2018). Factors influencing medical students' choice of emergency medicine as a career specialty-a descriptive study of Saudi medical students. Int J Emerg Med.

[22] Singh A, Alberti H (2021). Why UK medical students change career preferences: an interview study. Perspect Med Educ.

[23] Levaillant M, Levaillant L, Lerolle N, Vallet B, Hamel-Broza JF (2020). Factors influencing medical students' choice of specialization: A gender based systematic review. EClinicalMedicine.

[24] Alkhamees MA, Almutairi SA, Aljuhayman AM, Alkanhal H, Alenezi SH, Almuhaideb M, Alkhateeb SS (2021). Evaluation of the urology residency training program in Saudi Arabia: A cross-sectional study. Urol Ann.

[25] Neal MT, Lyons MK (2020). Burnout and work-life balance in neurosurgery: Current state and opportunities. Surg Neurol Int.

[26] Streu R, McGrath MH, Gay A, Salem B, Abrahamse P, Alderman AK (2011). Plastic surgeons' satisfaction with work-life balance: results from a national survey. Plast Reconstr Surg.

[27] Verret CI, Nguyen J, Verret C, Albert TJ, Fufa DT (2021). How do areas of work life drive burnout in orthopaedic attending surgeons, fellows, and residents?. Clin Orthop Relat Res.

